# Genetic Diversity of Aromatic Rice Germplasm Revealed By SSR Markers

**DOI:** 10.1155/2018/7658032

**Published:** 2018-03-15

**Authors:** Saba Jasim Aljumaili, M. Y. Rafii, M. A. Latif, Siti Zaharah Sakimin, Ibrahim Wasiu Arolu, Gous Miah

**Affiliations:** ^1^Laboratory of Climate-Smart Food Crop Production, Institute of Tropical Agriculture and Food Security, Universiti Putra Malaysia (UPM), 43400 Serdang, Selangor, Malaysia; ^2^Department of Crop Science, Faculty of Agriculture, Universiti Putra Malaysia (UPM), 43400 Serdang, Selangor, Malaysia; ^3^Bangladesh Rice Research Institute, Gazipur, Dhaka, Bangladesh; ^4^Agronomy Department, Crown Flour Mills, KM 25 Kaduna-Abuja Expressway, Kaduna, Nigeria

## Abstract

Aromatic rice cultivars constitute a small but special group of rice and are considered the best in terms of quality and aroma. Aroma is one of the most significant quality traits of rice, and variety with aroma has a higher price in the market. This research was carried out to study the genetic diversity among the 50 aromatic rice accessions from three regions (Peninsular Malaysia, Sabah, and Sarawak) with 3 released varieties as a control using the 32 simple sequence repeat (SSR) markers. The objectives of this research were to quantify the genetic divergence of aromatic rice accessions using SSR markers and to identify the potential accessions for introgression into the existing rice breeding program. Genetic diversity index among the three populations such as Shannon information index (*I*) ranged from 0.25 in control to 0.98 in Sabah population. The mean numbers of effective alleles and Shannon's information index were 0.36 and 64.90%, respectively. Similarly, the allelic diversity was very high with mean expected heterozygosity (*H*_*e*_) of 0.60 and mean Nei's gene diversity index of 0.36. The dendrogram based on UPGMA and Nei's genetic distance classified the 53 rice accessions into 10 clusters. Analysis of molecular variance (AMOVA) revealed that 89% of the total variation observed in this germplasm came from within the populations, while 11% of the variation emanated among the populations. These results reflect the high genetic differentiation existing in this aromatic rice germplasm. Using all these criteria and indices, seven accessions (Acc9993, Acc6288, Acc6893, Acc7580, Acc6009, Acc9956, and Acc11816) from three populations have been identified and selected for further evaluation before introgression into the existing breeding program and for future aromatic rice varietal development.

## 1. Introduction

Rice (*Oryza sativa* L.) is one of the most important food crops in the world. Approximately three billion people around the world consume rice as a basic food which provides about 50 to 80% of their daily calories. Aromatic rice is preferred over nonaromatic rice during special occasions and for export, and thus it commands a higher market price. One of the major features of this kind of rice is its special aroma that is appreciated by many people and represents a high value-added trait [1]. Three different things seem to have led to the growth in popularity of aromatic rice: globalization, health consciousness, and culinary changes [2]. Consequently, rice needs attention to the improvement of its cooking qualities as well as its several biochemical and morphological characteristics [3]. The demand for aromatic rice is increasing day by day. Unfortunately, aromatic rice production is affected by some abiotic and biotic stresses, susceptibility to pests and diseases, and strong shedding [4].

Agronomic value of rice variety depends on many characteristics [5]. The most important features include high yielding ability, resistance to diseases and pests, resistance to undesirable environmental factors, and high quality of the products. Genetic diversity studies occupy an important position in breeding and improvement program as they ensure efficient utilization of germplasm resources and effective breeding system for the improvement of closely related crop species. Genetic variation analysis helps breeders in observing germplasm as well as predicting possible genetic potentials [6]. The improvement of rice breeding plummeted progressively during the last ten years due to the poor basis of the parent materials [7]. The research of the rice genetic variety is essential for cultivars rating, identification, conservation, and purity as well as breeding [8].

Genetic diversity is mainly measured based on the morphological differences of quantitative important traits. However, this method has some disadvantages in terms of time, space, and labour cost. In addition, this method cannot define the exact level of genetic diversity among the germplasms, because of the additive gene action on the expression of the traits (economically important traits), thus making environmental factors mask their true phenotypic performance [9, 10]. Phenotypic expression is affected by the environment; therefore, selection based on morphological traits is seductive [11, 12]. Use of molecular markers is the chromosomal landmark through which an organism can be recognized and has gained popularity as a genetic diversity tool [13]. Among the PCR-based markers, the SSR markers have proved to be very effective tools in the study of genetic diversity and organism relationships due to their high polymorphic nature and transferability [13, 14]. In recent years, microsatellite markers have been widely used to screen, characterize, and evaluate genetic diversity in many crop species [15].

For marker-assisted selection as well as gene tagging, rice microsatellites had shown their utility [16, 17]. The SSR markers can be effectively applied for developing unique DNA profiles of rice genotypes because of having a high level of polymorphism and greater information. The statistical analysis has been used to measure the mutual relationships between various characters and yield improvement. Genotypic evaluation of yield components can identify their relationship with grain yield in aromatic rice and the information of these relationships can be helpful to find superior aromatic rice genotypes [18]. In the present study, the genetic diversity of several high yielding aromatic rice genotypes was determined by using SSR markers. This was needed to identify the potential diverse genotypes for use as a parent in future rice breeding program.

## 2. Materials and Methods

### 2.1. Plant Material and Experimental Design

A total of 53 rice accessions including three local check varieties (MRQ74, MR219, and MR253) were used in this study as shown in Table 1. These rice accessions and check varieties were collected from the Malaysian Agricultural Research and Development Institute (MARDI). These rice accessions were collected from Sarawak (10), Peninsular Malaysia (10), and Sabah (30) by MARDI. All of the accessions were* indica* type. The experiment was conducted inside the net house at the experimental field of Universiti Putra Malaysia. The sprouted seeds of the 53 rice accessions were sown in the different pots using randomized complete block design (RCBD) with three replications.

### 2.2. Selection of SSR Markers

A total of 147 SSR markers were selected for diversity analysis, out of which 32 primers showed clear, distinct polymorphic bands among the 53 aromatic rice accessions selected for the analysis as shown in Table 2.

### 2.3. DNA Extraction

The DNA was extracted from 21-day-old seedlings leaves of aromatic rice genotypes using hexadecyltrimethylammonium bromide (CTAB) method [19]. The quality of DNA was determined by running it on 1% agarose gel with 1x TBE buffer (Trizma base with EDTA and boric acid; pH was adjusted to 8.0 with NaOH) at 70 V for 45 minutes. The gel was observed by UV Transilluminator lamp. The assessment of DNA concentration was implemented using NanoDrop Spectrophotometer (ND-1000, NanoDrop Inc., USA). The DNA was diluted to 50 ng using TE buffer and stored at 4°C before for the commencement of PCR.

### 2.4. SSR PCR Protocol and Bands Separation

Polymorphic thirty-two (32) SSR markers were used for genotyping the entire 53 rice accessions. Total PCR reaction was optimized to be 15 *μ*l and this included 1 *μ*l of about 50 ng DNA template, 7.0 *μ*l DreamTaq PCR master mix (Thermos Scientific Inc.), 1 *μ*l of each primer (forward and reverse primer), and 5.0 *μ*l nuclease free water. Touch-down PCR protocol was followed [20]. The band separation was done by running the PCR products on 3% metaphor agarose at 80 v for 60 min in 1% TBE along with 50 bp DNA ladder. The gel was viewed using Bio-Rad gel documentation machine. The gel picture was analyzed using Bio-Rad Image lab software for the band size. The data were saved in Excel for further analysis.

### 2.5. Data Analysis

Genetic diversity parameters such as percentage polymorphic loci (PPL), effective allele number (*N*_*e*_), gene diversity (*h*), Shannon's information index (*I*), gene frequency, and gene flow (*N*_*m*_) were computed [21]. Genetic differentiation of population (GST), which is the measure of the proportional amount of variation within subpopulation as compared with the total population, was computed. When GST is equal to “0,” this implies that the subpopulations are identical; when the value is “1,” they are completely different. The gene flow was computed from GST as follows:

(1) $$\begin{array}{c} N_{m}=\frac{\left(1/4\right)\left(1-G_{st}\right)}{G_{s}}, \end{array}$$

where *N* is the effective population size and *m* is the fraction of individuals in a population. If *N*_*m*_ is <1, this indicates that populations tend to differentiate, and when *N*_*m*_ ≥ 1, there is little differentiation among populations. Polymorphism information content (PIC) was computed from the formula given below:

(2) $$\begin{array}{c} PIC=1-\overset{n}{\underset{j=1}{\sum }}P_{ij}^{2}, \end{array}$$

where *P*_*ij*_ is the frequency of the *j*th allele for the *i*th marker and summed over *n* alleles.

Analysis of molecular variance (AMOVA) was conducted to assess the genetic structure of the populations [22] using Arlequin software. Cluster analysis was performed to obtain dendrogram based on similarity coefficient using unweighted pair group method with arithmetic mean (UPGMA). Additionally, the covalence structure of the 53 accessions was determined through three-dimensional principal component analysis using NTSYS-pc software (version 2.1).

## 3. Results and Discussion

The present study evaluated the genetic diversity of 50 aromatic rice accessions and 3 check varieties as a control. These accessions were obtained from three different regions, namely, Sarawak, Peninsular Malaysia, and Sabah. The study of genetic diversity in any breeding population is essential as it constitutes the backbone of any breeding and improvement program. It helps in the development of crop that is suitable and adaptable to rapid climate change through the introduction of foreign genes [23, 24]. Thus, genetic diversity is needed for developing ideal and desired crop varieties for present and future needs. In this study, one hundred and forty-seven (147) SSR markers were screened, out of which 32 SSR markers were found to be polymorphic and suitable for diversity analysis. The use of SSR for rice diversity study is very crucial as it provides accurate and unbiased assessment and reveals in-depth information on the genetic divergence of a germplasm material [25]. SSR marker has been widely recognized for its codominant inheritance pattern, high informative power, and transferability among the species, hence, its superiority as a marker of choice for plant germplasm improvement program [26, 27].

### 3.1. Allelic Diversity

**Figure 1:**
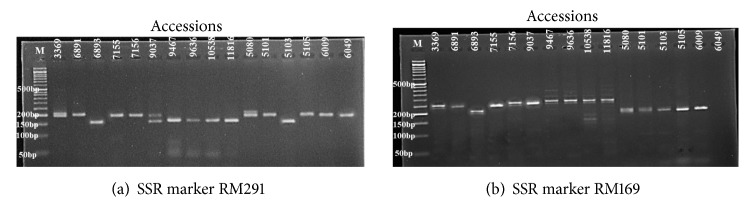
(a) Polymorphism among accessions using RM291 SSR marker, M ladder (50 bp). (b) Polymorphism among accessions using RM169 SSR marker, M ladder (50 bp).

From 147 SSR markers screened, 32 markers (21.77%), which displayed clear and repeatable polymorphic bands, were selected for analysis as shown in Table 3 and Figures 1(a) and 1(b). A total of 131 alleles were recorded, and the number of alleles per locus ranged from 2 in RM3134 to 7 in RM462 with an average of 4.09. The expected heterozygosity differed among the markers and it ranged from 0.01 (RM23) to 1.13 (RM172) with an average of 0.60.

PIC was observed to differs significantly, from 0.25 (RM3872) to 0.98 (RM321). The effective number of alleles (*N*_*e*_) varied from 0.10 (RM16655) to 2.12 (RM3134) with an average of 1.48. Nei's gene diversity (*h*) ranged from 0.05 (RM172) to 0.98 (RM1), with a mean of 0.36. Shannon's information index ranged from 0.22 in RM460 to 0.91 in RM195; the average was 0.58 as shown in Table 3.

Results from the present investigation revealed remarkably abundant genetic variation among the 53 aromatic rice genotypes. The number of alleles ranged from 2 to 7. The number of alleles observed in this study was higher than findings of [28, 29], where the alleles' number was reported within 2.40 to 3.35 per locus. On the other hand, higher number of alleles as much as 6.60 to 14.60 have been reported using other rice varieties [30, 31]. A total of 128 alleles with an average of 3.28 alleles per locus and PIC value of 0.24 were observed by [32] using 39 SSR markers.

The number of alleles indicates the richness of the population. Since SSR are short tandem repeats, generally allele numbers of 2 to 7 alleles per locus are considered good as seen in this study. Allele number of 1–6 alleles/locus with an average of 3.24 has been reported in colored upland rice germplasm in Malaysia [25]. The PIC value ranged from 0.25 to 0.98 with an average of 0.61. The richness of information a marker can give, otherwise known as PIC reported in this study, was very interesting. Similarly, lower genetic diversity was reported with an average of 2.75 alleles per locus and an average PIC value of 0.38 from 40 Pakistan rice accessions [33]. On the other hand, 4.69 alleles per locus were observed with an average PIC value of 0.81 among the 36 landraces having different therapeutic values from India [34].

High PIC of 0.25 to 0.98 as seen in this study revealed that the markers have the required properties to be used in diversity study. The average PIC value in this study was higher than the value from [35] that reported average PIC value equivalent to 0.39. Genetic diversity indices such as expected heterozygosity as well as Shannon's and Nei's index among the markers were very high (>0.5, except in Nei's index), thus reflecting the heterozygous nature of the population. Percentages of polymorphic bands for Sarawak, Peninsular Malaysia, Sabah, and check groups were 75.66, 66.07, 95.46, and 22.40%, respectively, with an average of 64.90%, as shown in Table 4. Among the populations, Sabah population exhibited highest genetic diversity levels (95.46%), while control varieties had the lowest genetic diversity (22.40%).

The average number of alleles per locus (*N*_*a*_) varied from 0.80 (control) to 1.98 (Sabah). The effective number of alleles per locus (*N*_*e*_) was small compared to the number of alleles per locus and it ranged from 0.16 (control) to 0.54 (Sabah) with the mean number of 0.36. Shannon's information index (*I*) was very high and varied from 0.25 (control) to 0.98 (Sabah) with 0.58 as mean as shown in Table 4. High and low genetic diversity index as seen in the Sabah and control population might not be unconnected with the population size. Population size plays an important role in genetic differentiation in germplasm. The higher the population is, the more the likelihood of genetic differentiation is and, thus, the higher the heterozygosity is [36]. The magnitude of the stochastic process and the degree of change in genetic properties of a population depend on its effective size [37].

Additionally, Shannon's information index, another population genetic parameter, ranged from 0.25 for control varieties to 0.98 for Sabah accessions with an average of 0.58. This average value was less than the value described earlier [38], which was found to be 0.88. The high value of Shannon's information index in the present study was another indication of the presence of high genetic diversity in the rice germplasm under consideration. Nei's gene diversity, which varied from 0.16 for check accessions to 0.58 for Sabah varieties with an average value of 0.36, also indicated the high level of divergence in the population. This value aligned with that of [39], which reported mean Nei's gene diversity of 0.37 but lower than 0.50 [40].

### 3.2. Cluster Analysis and Principal Component Analysis

**Figure 2:**
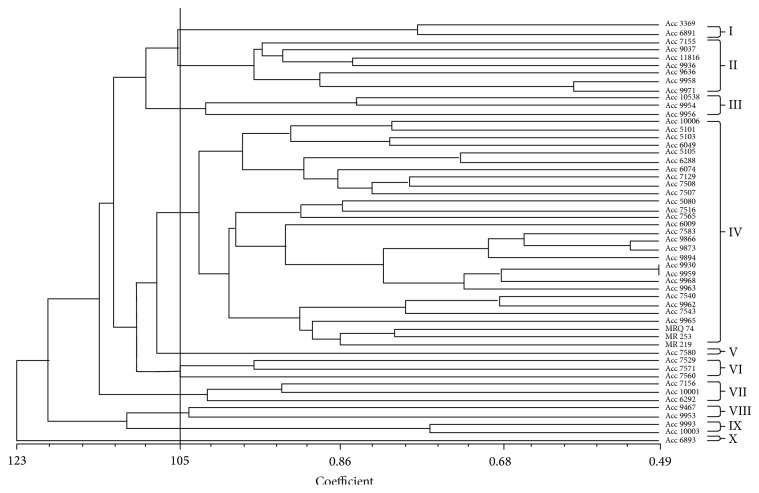
Cluster analysis of 53 accessions of aromatic rice based on SSR markers polymorphism.

Cluster analysis based on UPGMA method grouped the 53 accessions into ten distinct clusters at the coefficient of 1.05. The distance coefficient ranged from 0.49 to 1.23 as shown in Figure 2. Cluster I consisted of 2 accessions, while clusters II and III had 7 and 3 accessions, respectively. Cluster IV had the highest number of accessions with 29 rice accessions. Clusters V, VI, VII, VIII, IX, X had 1, 3, 3, 2, 2, 1, respectively, as shown in Table 5. Cluster II comprises accessions mostly of Sarawak (4) and Sabah (3) origin, while cluster IV comprises entire check varieties (3) together with all the accessions from Peninsular Malaysia except one accession (Acc6292). Additionally, 17 out of 30 accessions of Sabah origin were also found in cluster IV. Accessions from Sabah population were found virtually in the cluster group except for clusters I and II which were found to be lacking.

**Figure 3:**
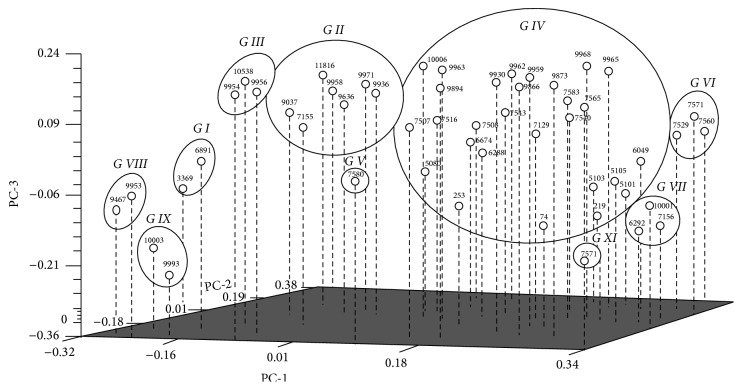
Three-dimensional PCA of 53 aromatic rice accessions based on 32 SSR markers.

Critical analysis of the covariance displacement and structure as revealed by the three-dimensional PCA shows that 53 accessions were clustered into 10 distinct groups, corroborating the output from cluster analysis as shown in Figure 3. Groups V and X were found to have one accession each, while groups I, VIII, and IX contain 2 accessions each. On the other hand, groups III, VI, and VII had three members each, while groups II and IV were found to be exceptional in terms of high membership with 7 and 29 rice accessions, respectively. The result of PCA showed clear geographical correspondence to the accession with grouping patterns.

In a previous study, 29 rice genotypes were reported to have been grouped into two distinct clusters with the aid of 20 SSR markers [41]. In another study, three distinct clusters were also found from 18 rice cultivars studied using 44 SSR markers [39]. The clustering patterns in the current study gave some consideration to the geographical origin of the populations. As evident from the dendrogram, all the accessions in cluster I came from Sarawak population, while four out of seven (7) accessions from cluster II belonged to Sarawak population. Additionally, all the check varieties which were used as the control population were grouped together in cluster IV.

All these point to the accuracy and usefulness of the SSR markers in tracing the phylogeny or pedigree of a germplasm or breeding materials. This observation corresponds to the previous observations of other rice germplasm studies [42, 43]. In another rice diversity study, 42 colored rice varieties were reported to have clustered according to their country and region of origin [25]. Similarly, clustering pattern has also been reported by [44] based on allelic and morphological data along with the location in rice varieties using SSR markers. Accessions that are found clustered together are assumed to have high genetic similarity, while those that are found far away from each other are considered to be divergent.

### 3.3. Analysis of Molecular Variance

The AMOVA results displayed highly significant genetic differences among accessions within the population. Of the total genetic variation in the 53 accessions, 89% was due to genetic variation within the population. This indicates the existence of high genetic differentiation among the genotypes within the groups. On the other hand, the genetic variation among the group accounted for 11% of the total variation as shown in Table 6. Consequently, the differential between the overall groups and their geographical groups had really happened and resulted in relatively high genetic diversity.

Variation of similar pattern as observed in this study among rice germplasm has been reported in a previous study [45]. In one study, which involved 41 rice genotypes from three populations, 67% of the total variation was attributed to variation within the genotype while variation among the three populations accounted for the remaining 33%. The presence of high variability within the population represents high level of genetic differentiation which will further strengthen the divergence of the population. High genetic differentiation is very important within the germplasm for creating a desirable heterotic group in base breeding populations [23]. Thus genetic diversity characterization is very important as it provides the basis for planning conservation strategy, utilization, and establishment of breeding and improvement for rice plant [46].

## 4. Conclusions

Genetic diversity is an important concept in any breeding program. It can be studied using SSR markers for the identification of potential parent in order to achieve heterosis in future aromatic rice breeding program. SSR markers were exploited to provide an unbiased estimate of the diversity pattern in this rice germplasm. The current study found the existence of high levels of diversity among 53 rice accessions which are good for the introduction of new genes in the existing genotypes. The dendrogram constructed to identify the genetic similarities among these genotypes showed that accessions from the same regions were found to cluster mostly together implying a correlation between molecular groupings and their source of collection. Clustering pattern on the basis of SSR markers provides ample information in identification and confirmation of rice genotypes and accessions. This information is significantly crucial for the development of pure aromatic rice breeding lines. Rice genotypes sharing common sources clustered into the same group. Based on SSR diversity analysis and clustering patterns, the following accessions (Acc9993, Acc6288, Acc6893, Acc7580, Acc6009, Acc9956, and Acc11816) have been identified as diverse accessions and suitable as a parent in the future breeding program.

## Figures and Tables

**Table 1:** Origin and local names of the aromatic rice accessions.

Sl. no.	Accession no.	Origin	Local name
(1)	Acc3369	Sarawak	Mansau
(2)	Acc6891	Sarawak	Biris
(3)	Acc6893	Sarawak	Padi Wangi
(4)	Acc7155	Sarawak	Chelom I
(5)	Acc7156	Sarawak	Chendana
(6)	Acc9037	Sarawak	Alek
(7)	Acc9467	Sarawak	Padi Chelum
(8)	Acc9636	Sarawak	Padi Wangi
(9)	Acc10538	Sarawak	Wangi
(10)	Acc11816	Sarawak	Padi Cigarette
(11)	Acc5080	Peninsular Malaysia	Chempa (Padi Huma)
(12)	Acc5101	Peninsular Malaysia	Siong Pelandok
(13)	Acc5103	Peninsular Malaysia	Anak Cina (H)
(14)	Acc5105	Peninsular Malaysia	Bongkok
(15)	Acc6009	Peninsular Malaysia	Mayang Lega
(16)	Acc6049	Peninsular Malaysia	Putih (Padi Huma)
(17)	Acc6288	Peninsular Malaysia	Chendana Wangi
(18)	Acc6292	Peninsular Malaysia	Santap Wangi
(19)	Acc6674	Peninsular Malaysia	Kuku Belang
(20)	Acc7129	Peninsular Malaysia	Rambut
(21)	Acc9936	Sabah	Janda Muda
(22)	Acc9953	Sabah	Padi Purak
(23)	Acc9954	Sabah	Padi Mansud
(24)	Acc9956	Sabah	Padi Beruang
(25)	Acc9958	Sabah	Padi Tiga Bulan
(26)	Acc9971	Sabah	Padi Pengalaan
(27)	Acc9993	Sabah	Liwagu Antap
(28)	Acc10001	Sabah	Padi Adong
(29)	Acc10003	Sabah	Padi Tubowan
(30)	Acc10006	Sabah	Padi Lasui
(31)	Acc7507	Sabah	Nangka
(32)	Acc7508	Sabah	Beliong
(33)	Acc7516	Sabah	Nangka2
(34)	Acc7529	Sabah	Kambaring A
(35)	Acc7540	Sabah	Putus Tunang
(36)	Acc7543	Sabah	Semilai
(37)	Acc7560	Sabah	Pagalan
(38)	Acc7565	Sabah	Kungkuling
(39)	Acc7571	Sabah	Turayan
(40)	Acc7580	Sabah	Kerikis
(41)	Acc7583	Sabah	Kedinga
(42)	Acc9866	Sabah	Padi Kolomintuhon
(43)	Acc9873	Sabah	Padi Mayang
(44)	Acc9894	Sabah	Dihangkang Putih
(45)	Acc9930	Sabah	Padi Solung
(46)	Acc9959	Sabah	Gebokong
(47)	Acc9962	Sabah	Tahi Ayam
(48)	Acc9963	Sabah	Padi Emas
(49)	Acc9965	Sabah	Padi Porak/Padi Taun
(50)	Acc9968	Sabah	Padi Susiah
(51)	MRQ74	Control	Check variety
(52)	MR219	Control	Check variety
(53)	MR253	Control	Check variety

**Table 2:** Selected polymorphic simple sequence repeat markers.

Sl. no.	SSR marker	Sequence	Tem (°C)	Chr. no.
(1)	RM1	F: 5′-GCGAAAACACAATGCAAAAA -3′	55	1
		R: 5′-GCGTTGGTTGGACCTGAC-3′		
(2)	RM23	F: 5′-CATTGGAGTGGAGGCTGG-3′	55	1
		R: 5′-GTCAGGCTTCTGCCATTCTC-3′		
(3)	RM38	F: 5′- ACGAGCTCTCGATCAGCCTA-3′	55	8
		R: 5′- TCGGTCTCCATGTCCCAC-3′		
(4)	RM108	F: 5′-TCTCTTGCGCGCACACTGGCAC-3′	67	9
		R: 5′-CGTGCACCACCACCACCACCAC-3′		
(5)	RM114	F: 5′-CAGGGACGAATCGTCGCCGGAG-3′	55	3
		R: 5′-TTGGCCCCCTTGAGGTTGTCGG-3′		
(6)	RM159	F: 5′-GGGGCACTGGCAAGGGTGAAGG-3′	55	5
		R: 5′-GCTTGTGCTTCTCTCTCTCTCTCTCTCTC-3′		
(7)	RM165	F: 5′-CCGAACGCCTAGAAGCGCGTCC-3′	67	1
		R: 5′-CGGCGAGGTTTGCTAATGGCGG-3′		
(8)	RM169	F: 5′-TGGCTGGCTCCGTGGGTAGCTG -3′	67	5
		R: 5′-TCCCGTTGCCGTTCATCCCTCC-3′		
(9)	RM172	F: 5′-TGCAGCTGCGCCACAGCCATAG-3′	55	7
		R: 5′- CAACCACGACACCGCCGTGTTG-3′		
(10)	RM195	F: 5′-AGAAAGAGAGGCCGTCGGCGGC-3′	61	8
		R: 5′-GGGCTCACCCCCAAACCTGCAG-3′		
(11)	RM250	F: 5′- GGTTCAAACCAAGCTGATCA-3′	55	2
		R: 5′- GATGAAGGCCTTCCACGCAG-3′		
(12)	RM256	F: 5′-GACAGGGAGTGATTGAAGGC-3′	55	8
		R: 5′-GTTGATTTCGCCAAGGGC-3′		
(13)	RM285	F: 5′-CTGTGGGCCCAATATGTCAC-3′	55	9
		R: 5′-GGCGGTGACATGGAGAAAG-3′		
(14)	RM288	F: 5′-CCGGTCAGTTCAAGCTCTG-3′	55	9
		R: 5′-ACGTACGGACGTGACGAC-3′		
(15)	RM291	F: 5′-GTTGCACTACGTATTCTGAG-3′	55	5
		R: 5′-GATCCAGATAAATGAGGCAC-3′		
(16)	RM294	F: 5′-TTGGCCTAGTGCCTCCAATC-3′	55	1
		R: 5′-GAGGGTACAACTTAGGACGCA-3′		
(17)	RM311	F: 5′-TGGTAGTATAGGTACTAAACAT-3′	55	10
		R: 5′-TCCTATACACATACAAACATAC-3′		
(18)	RM314	F: 5′-CTAGCAGGAACTCCTTTCAGG-3′	55	6
		R: 5′-AACATTCCACACACACACGC		
(19)	RM321	F: 5′-CCAACACTGCCACTCTGTTC-3′	55	9
		R: 5′-GAGGATGGACACCTTGATCG-3′		
(20)	RM327	F: 5′-CTACTCCTCTGTCCCTCCTCTC-3′	55	2
		R: 5′-CCAGCTAGACACAATCGAGC-3′		
(21)	RM332	F: 5′-GCGAAGGCGAAGGTGAAG -3′	55	11
		R: 5′-CATGAGTGATCTCACTCACCC-3′		
(22)	RM342	F: 5′-CCATCCTCCTACTTCAATGAAG-3′	55	8
		R: 5′-ACTATGCAGTGGTGTCACCC-3′		
(23)	RM434	F: 5′-GCCTCATCCCTCTAACCCTC-3′	55	9
		R:5′-CAAGAAAGATCAGTGCGTGG-3′		
(24)	RM460	F: 5′-TGATCGACAGCGTTCTTGAC-3′	55	9
		R: 5′-GCCTGGCCCACATAATTAAG-3′		
(25)	RM469	F: 5′-AGCTGAACAAGCCCTGAAAG-3′	55	6
		R: 5′-GACTTGGGCAGTGTGACATG-3′		
(26)	RM3134	F: 5′-GCAGGCACAAAAGCAAAGAG-3′	50	3
		R: 5′-AGGTGAAGGTGCATTGTGTG-3′		
(27)	RM3872	F: 5′-GGAAGAAAGGATCTATATCA-3′	55	3
		R:5′-TACGATTTGTTTAAGTTCAA-3′		
(28)	RM6250	F: 5′-AACCTACGTTACCCTGCACG-3′	50	4
		R: 5′-GGCTCATGAGTTTCAGAGGC-3′		
(29)	RM7376	F: 5′-TCACCGTCACCTCTTAAGTC-3′	50	12
		R: 5′-GGTGGTTGTGTTCTGTTTGG-3′		
(30)	RM10022	F: 5′-CCTCCATAGAGTAAGGTTTGCATGG-3′	50	1
		R: 5′-CCTCCTCCTCTGTCTTTCTCTGC-3′		
(31)	RM16655	F: 5′-CCTTGGAAGCTGGAACTTCACC-3′	50	4
		R: 5′-GGCTCTTAGGTTAGATCCCACACG-3′		
(32)	RM23835	F: 5′-TTCCGCTGTTTCTCTTCTTGTGC-3′	50	9
		R: 5′-CTGGTTCTGCTGGTTCTGTAGTTGG-3′		

**Table 3:** Allelic diversity as revealed by 32 polymorphic SSR markers.

Sl. no.	Marker	Number of alleles	Expected heterozygosity	PIC	Effective no. of alleles	Nei's index	Shannon's index
(1)	RM1	5	0.35	0.87	1.91	0.98	0.84
(2)	RM23	6	0.01	0.76	1.57	0.36	0.67
(3)	RM38	5	0.37	0.46	1.70	0.27	0.47
(4)	RM108	4	0.51	0.45	1.62	0.38	0.74
(5)	RM114	5	0.68	0.58	1.09	0.43	0.73
(6)	RM159	6	0.53	0.78	1.99	0.50	0.69
(7)	RM165	5	0.77	0.40	1.53	0.35	0.53
(8)	RM169	3	0.73	0.47	1.65	0.39	0.58
(9)	RM172	3	1.13	0.99	1.05	0.05	0.53
(10)	RM195	3	0.67	0.91	1.72	0.42	0.91
(11)	RM250	4	0.56	0.35	0.97	0.22	0.64
(12)	RM256	3	0.36	0.51	1.52	0.47	0.60
(13)	RM285	4	0.50	0.25	0.98	0.19	0.53
(14)	RM288	3	0.48	0.40	1.54	0.35	0.66
(15)	RM291	4	0.79	0.54	1.76	0.43	0.76
(16)	RM294	4	0.59	0.93	1.64	0.39	0.58
(17)	RM311	4	0.59	0.76	1.99	0.50	0.69
(18)	RM314	5	0.71	0.76	1.74	0.38	0.48
(19)	RM321	4	0.44	0.98	1.35	0.26	0.42
(20)	RM327	5	0.53	0.49	1.24	0.46	0.51
(21)	RM332	3	0.44	0.37	1.49	0.33	0.51
(22)	RM342	5	0.67	0.74	1.08	0.13	0.60
(23)	RM460	3	0.94	0.30	1.12	0.11	0.22
(24)	RM462	7	0.75	0.75	1.76	0.28	0.32
(25)	RM469	3	0.55	0.70	1.98	0.50	0.69
(26)	RM3134	3	0.48	0.78	2.12	0.70	0.82
(27)	RM3872	2	0.45	0.25	1.29	0.23	0.47
(28)	RM6250	4	0.55	0.79	1.84	0.37	0.41
(29)	RM7376	3	0.95	0.89	0.89	0.13	0.61
(30)	RM10022	4	0.67	0.55	1.63	0.34	0.54
(31)	RM16655	5	0.70	0.64	0.10	0.32	0.45
(32)	RM23835	4	0.85	0.35	1.36	0.41	0.37

Total no. of alleles: 131							

Mean		**4.09**	**0.60**	**0.63**	**1.48**	**0.36**	**0.58**

**Table 4:** Genetic diversity parameters based on the rice populations.

Group	PIC	*N*_*a*_	*N*_*e*_	*I*
Sarawak	75.66	1.59	0.37	0.61
Peninsular Malaysia	66.07	1.58	0.33	0.46
Sabah	95.46	1.98	0.58	0.98
Control	22.40	0.80	0.16	0.25

Mean	64.90	1.49	0.36	0.58

PIC: percentages of polymorphic bands (%); *N*_*a*_: average number of alleles per locus. *N*_*e*_: the effective number of alleles per locus. *I*: Shannon's information index.

**Table 5:** Cluster group based on 32 SSR markers.

Cluster	Number of accessions	Accessions
I	2	Acc3369, Acc6891

II	7	Acc7155, Acc9037, Acc11816, Acc9936, Acc9636, Acc9958, Acc9971

III	3	Acc10538, Acc9954, Acc9956

IV	29	Acc10006, Acc5101, Acc5103, Acc6049, Acc5105, Acc6288, Acc6674, Acc7129, Acc7508, Acc7507, Acc5080, Acc7516, Acc7565, Acc6009, Acc7583, Acc9866, Acc9873, Acc9894, Acc9930, Acc9959, Acc9968, Acc9963, Acc7540, Acc9962, Acc7543, Acc9965, MRQ74, MR253, MR219.

V	1	Acc7580

VI	3	Acc7529, Acc7571, Acc7560

VII	3	Acc7156, Acc10001, Acc6292

VIII	2	Acc9467, Acc9953

IX	2	Acc9993, Acc10003

X	1	Acc6893

**Table 6:** Analysis of molecular variance for 53 rice accessions.

Source	df	SS	MS	Variance component	% of variation
Among populations	3	283.40	84.82	4.30	11
Within populations	49	1688.16	32.39	33.29	89
Total	52	1971.56		37.99	100

df: degree of freedom; SS: sum of squares; MS: mean squares.

## Supplementary Materials

Supplementary Materials All the 147 SSR markers that were used in this study are presented in a supplementary table.
