# Supplementary material for: Genetic Diversity of Aromatic Rice Germplasm Revealed By SSR Markers
Source: Biomed Res Int. 2018 Mar 15;2018:7658032. doi: 10.1155/2018/7658032 (PMC5874984; doi:10.1155/2018/7658032)
Supplement: Supplementary Materials — All the 147 SSR markers that were used in this study are presented in a supplementary table. [file 7658032.f1.docx]

**Supplementary Table: List of 147 SSR markers examined under this study**

| **Serial No.** | **SSR Marker** | **Sequence** | **Tm (°C)** |
| --- | --- | --- | --- |
| 1 | RM1 | F: 5′-GCGAAAACACAATGCAAAAA -3′ | 55 |
|  |  | R:5′-GCGTTGGTTGGACCTGAC-3′ |  |
| 2 | RM7 | F: 5′-TTCGCCATGAAGTCTCTCG-3′ | 55 |
|  |  | R:5′-CCTCCCATCATTTCGTTGTT-3′ |  |
| 3 | RM8 | F: 5′-CACGTGGCGTAAATACACGT-3′ | 55 |
|  |  | R:5′-GGCCAAACCCTAACCCTG-3′ |  |
| 4 | RM10 | F: 5′-TTGTCAAGAGGAGGCATCG-3′ | 55 |
|  |  | R:5′-TTGTCAAGAGGAGGCATCG-3′ |  |
| 5 | RM13 | F: 5′-TCCAACATGGCAAGAGAGAG-3′ | 55 |
|  |  | R:5′-GGTGGCATTCGATTCCAG -3′ |  |
| 6 | RM21 | F: 5′-ACAGTATTCCGTAGGCACGG -3′ | 55 |
|  |  | R:5′-GCTCCATGAGGGTGGTAGAG-3′ |  |
| 7 | RM23 | F: 5′-CATTGGAGTGGAGGCTGG-3′ | 55 |
|  |  | R:5′-GTCAGGCTTCTGCCATTCTC-3′ |  |
| 8 | RM25 | F: 5′-GGAAAGAATGATCTTTTCATGG-3′ | 55 |
|  |  | R:5′-CTACCATCAAAACCAATGTTC-3′ |  |
| 9 | RM38 | F: 5′- ACGAGCTCTCGATCAGCCTA-3′ | 55 |
|  |  | R: 5′- TCGGTCTCCATGTCCCAC-3′ |  |
| 10 | RM44 | F: 5′- ACGGGCAATCCGAACAACC-3′ | 55 |
|  |  | R: 5′- TCGGGAAAACCTACCCTACC-3′ |  |
| 11 | RM55 | F: 5′-CCGTCGCCGTAGTAGAGAAG-3′ |  |
|  |  | R:5′-TCCCGGTTATTTTAAGGCG-3′ |  |
| 12 | RM72 | F: 5′- CCGGCGATAAAACAATGAG-3′ | 55 |
|  |  | R: 5′- GCATCGGTCCTAACTAAGGG-3′ |  |
| 13 | RM82 | F: 5′- TGCTTCTTGTCAATTCGCC-3′ | 55 |
|  |  | R: 5′- CGACTCGTGGAGGTACGG-3′ |  |
| 14 | RM101 | F: 5′-GTGAATGGTCAAGTGACTTAGGTGGC-3′ | 55 |
|  |  | R: 5′-ACACAACATGTTCCCTCCCATGC-3′ |  |
| 15 | RM104 | F: 5′- GGAAGAGGAGAGAAAGATGTGTGTCG-3′ | 54 |
|  |  | R: 5′- TCAACAGACACACCGCCACCGC-3′ |  |
| 16 | RM106 | F: 5′- CGTCTTCATCATCGTCGCCCCG-3′ | 55 |
|  |  | R: 5′- GGCCCATCCCGTCGTGGATCTC-3 |  |
| 17 | RM107 | F: 5′-AGATCGAAGCATCGCGCCCGAG-3′ | 55 |
|  |  | R:5′-ACTGCGTCCTCTGGGTTCCCGG-3′ |  |
| 18 | RM108 | F: 5′-TCTCTTGCGCGCACACTGGCAC-3′ | 55 |
|  |  | R:5′-CGTGCACCACCACCACCACCAC-3′ |  |

**Appendix 4: Continued**

| **Serial No.** | **SSR Marker** | **Sequence** | **Tm (°C)** |
| --- | --- | --- | --- |
| 19 | RM109 | F: 5′-GCCGCCGGAGAGGGAGAGAGAG-3′ | 55 |
|  |  | R:5′-CCCCGACGGGATCTCCATCGTC-3′ |  |
| 20 | RM114 | F: 5′-CAGGGACGAATCGTCGCCGGAG-3′ | 55 |
|  |  | R:5′-TTGGCCCCCTTGAGGTTGTCGG-3′ |  |
| 21 | RM120 | F: 5′- CACACAAGCCCTGTCTCACGACC-3′ | 55 |
|  |  | R: 5′- CGCTGCGTCATGAGTATGTA-3 |  |
| 22 | RM136 | F: 5′-GAGAGCTCAGCTGCTGCCTCTAGC-3′ | 55 |
|  |  | R:5′-GAGGAGCGCCACGGTGTACGCC-3′ |  |
| 23 | RM142 | F: 5′- CTCGCTATCGCCATCGCCATCG-3′ | 67 |
|  |  | R: 5′- TCGAGCCATCGCTGGATGGAGG-3′ |  |
| 24 | RM153 | F: 5′-GCCTCGAGCATCATCATCAG -3′ | 55 |
|  |  | R:5′-ATCAACCTGCACTTGCCTGG -3′ |  |
| 25 | RM159 | F: 5′-GGGGCACTGGCAAGGGTGAAGG-3′ | 55 |
|  |  | R:5′-GCTTGTGCTTCTCTCTCTCTCTCTCTCTC-3′ |  |
| 26 | RM165 | F: 5′-CCGAACGCCTAGAAGCGCGTCC-3′ | 67 |
|  |  | R:5′-CGGCGAGGTTTGCTAATGGCGG-3′ |  |
| 27 | RM166 | F: 5′-GGTCCTGGGTCAATAATTGGGTTACC-3′ | 61 |
|  |  | R:5′-TTGCTGCATGATCCTAAACCGG-3′ |  |
| 28 | RM168 | F: 5′-TGCTGCTTGCCTGCTTCCTTT-3′ | 55 |
|  |  | R:5′-GAAACGAATCAATCCACGGC-3′ |  |
| 29 | RM169 | F: 5′-TGGCTGGCTCCGTGGGTAGCTG -3′ | 67 |
|  |  | R:5′-TCCCGTTGCCGTTCATCCCTCC-3 |  |
| 30 | RM172 | F: 5′-TGCAGCTGCGCCACAGCCATAG-3′ | 55 |
|  |  | R:5′- CAACCACGACACCGCCGTGTTG-3′ |  |
| 31 | RM177 | F: 5′- CCCTCTTAGACAGAGGCCAGAGGG-3′ | 61 |
|  |  | R: 5′- GTAGCCGAAGATGAGGCCGCCG-3′ |  |
| 32 | RM179 | F: 5′- CCCCATTAGTCCACTCCACCACC-3′ | 55 |
|  |  | R: 5′- CCAATCAGCCTCATGCCTCCCC-3′ |  |
| 33 | RM185 | F: 5′- AGTTGTTGGGAGGGAGAAAGGCC-3′ | 61 |
|  |  | R: 5′- AGGAGGCGACGGCGATGTCCTC-3′ |  |
| 34 | RM192 | F: 5′-GCGGCGGATCATGAATTGCGAG-3′ | 61 |
|  |  | R:5′-CTTGTTCCCCGGCGTCGAGTCC-3′ |  |
| 35 | RM195 | F: 5′-AGAAAGAGAGGCCGTCGGCGGC-3′ | 61 |
|  |  | R:5′-GGGCTCACCCCCAAACCTGCAG-3′ |  |
| 36 | RM206 | F: 5′-CCCATGCGTTTAACTATTCT-3′ | 55 |
|  |  | R:5′-CGTTCCATCGATCCGTATGG -3′ |  |

**Appendix 4: Continued**

| **Serial No.** | **SSR Marker** | **Sequence** | **Tm (°C)** |
| --- | --- | --- | --- |
| 37 | RM208 | F: 5′-TCTGCAAGCCTTGTCTGATG-3′ | 55 |
|  |  | R:5′-TAAGTCGATCATTGTGTGGACC-3′ |  |
| 38 | RM213 | F: 5′- ATCTGTTTGCAGGGGACAAG-3′ | 55 |
|  |  | R: 5′- AGGTCTAGACGATGTCGTGA-3′ |  |
| 39 | RM222 | F: 5′-CTTAAATGGGCCACATGCG-3′ | 55 |
|  |  | R:5′-CAAAGCTTCCGGCCAAAAG-3′ |  |
| 40 | RM223 | F: 5′-GAGTGAGCTTGGGCTGAAAC-3′ | 55 |
|  |  | R:5′-GAAGGCAAGTCTTGGCACTG-3′ |  |
| 41 | RM228 | F: 5′-CTGGCCATTAGTCCTTGG-3′ | 55 |
|  |  | R:5′-GCTTGCGGCTCTGCTTAC-3′ |  |
| 42 | RM231 | F: 5′-CCAGATTATTTCCTGAGGTC-3′ | 55 |
|  |  | R:5′-CACTTGCATAGTTCTGCATTG-3′ |  |
| 43 | RM232 | F: 5′-CCGGTATCCTTCGATATTGC-3′ | 55 |
|  |  | R:5′-CCGACTTTTCCTCCTGACG-3′ |  |
| 44 | RM233 | F: 5′-CCAAATGAACCTACATGTTG-3′ | 55 |
|  |  | R:5′-GCATTGCAGACAGCTATTGA-3′ |  |
| 45 | RM237 | F: 5′-CAAATCCCGACTGCTGTCC-3′ | 55 |
|  |  | R:5′-TGGGAAGAGAGCACTACAGC-3′ |  |
| 46 | RM244 | F: 5′-CCGACTGTTCGTCCTTATCA -3′ | 55 |
|  |  | R:5′-CTGCTCTCGGGTGAACGT-3′ |  |
| 47 | RM245 | F: 5′-ATGCCGCCAGTGAATAGC-3′ | 55 |
|  |  | R:5′-CTGAGAATCCAATTATCTGGGG-3′ |  |
| 48 | RM247 | F: 5′-TAGTGCCGATCGATGTAACG -3′ | 55 |
|  |  | R:5′-CATATGGTTTTGACAAAGCG -3′ |  |
| 49 | RM250 | F: 5′- GGTTCAAACCAAGCTGATCA-3′ | 55 |
|  |  | R: 5′- GATGAAGGCCTTCCACGCAG-3′ |  |
| 50 | RM256 | F: 5′-GACAGGGAGTGATTGAAGGC-3′ | 55 |
|  |  | R:5′-GTTGATTTCGCCAAGGGC-3′ |  |
| 51 | RM263 | F: 5′-CCCAGGCTAGCTCATGAACC-3′ | 55 |
|  |  | R:5′-GCTACGTTTGAGCTACCACG -3′ |  |
| 52 | RM274 | F: 5′-CCTCGCTTATGAGAGCTTCG-3′ | 55 |
|  |  | R:5′-CTTCTCCATCACTCCCATGG-3′ |  |
| 53 | RM284 | F: 5′-ATCTCTGATACTCCATCCATCC-3′ | 55 |
|  |  | R:5′-CCTGTACGTTGATCCGAAGC-3′ |  |
| 54 | RM285 | F: 5′-CTGTGGGCCCAATATGTCAC-3′ | 55 |
|  |  | R:5′-GGCGGTGACATGGAGAAAG-3′ |  |

**Appendix 4: Continued**

| **Serial No.** | **SSR Marker** | **Sequence** | **Tm (°C)** |
| --- | --- | --- | --- |
| 55 | RM288 | F: 5′-CCGGTCAGTTCAAGCTCTG-3′ |  |
|  |  | R:5′-ACGTACGGACGTGACGAC-3′ | 55 |
| 56 | RM291 | F: 5′-GTTGCACTACGTATTCTGAG-3′ | 55 |
|  |  | R:5′-GATCCAGATAAATGAGGCAC-3′ |  |
| 57 | RM294 | F: 5′-TTGGCCTAGTGCCTCCAATC-3′ | 55 |
|  |  | R:5′-GAGGGTACAACTTAGGACGCA-3′ |  |
| 58 | RM296 | F: 5′-CACATGGCACCAACCTCC-3′ | 55 |
|  |  | R:5′-GCCAAGTCATTCACTACTCTGG-3′ |  |
| 59 | RM301 | F: 5′-TTACTCTTTGTGTGTGTGTGAG-3′ | 55 |
|  |  | R:5′-CTACGACACGTCATAGATGACC-3′ |  |
| 60 | RM303 | F: 5′-GCATGGCCAAATATTAAAGG-3′ | 55 |
|  |  | R:5′-GGTTGGAAATAGAAGTTCGGT-3′ |  |
| 61 | RM305 | F: 5′-TACTGCCAAAGGCGAGCTTC-3′ | 55 |
|  |  | R:5′-GTGAGAGGCTACAGCTAACC-3′ |  |
| 62 | RM308 | F: 5′-GGCTGCACACGCACACTATA-3′ | 55 |
|  |  | R:5′-TTACGCATATGGTGAGTAGGC-3′ |  |
| 63 | RM311 | F: 5′-TGGTAGTATAGGTACTAAACAT-3′ | 55 |
|  |  | R:5′-TCCTATACACATACAAACATAC-3′ |  |
| 64 | RM312 | F: 5′-GTATGCATATTTGATAAGAG -3′ | 55 |
|  |  | R:5′-AAGTCACCGAGTTTACCTTC -3′ |  |
| 65 | RM314 | F: 5′-CTAGCAGGAACTCCTTTCAGG-3′ | 55 |
|  |  | R:5′-AACATTCCACACACACACGC |  |
| 66 | RM315 | F: 5′-GAGGTACTTCCTCCGTTTCAC-3′ | 55 |
|  |  | R:5′-AGTCAGCTCACTGTGCAGTG-3′ |  |
| 67 | RM317 | F: 5′-CATACTTACCAGTTCACCGCC-3′ | 55 |
|  |  | R:5′-CTGGAGAGTGTCAGCTAGTTGA-3′ |  |
| 68 | RM319 | F: 5′-ATCAAGGTACCTAGACCACCAC-3′ | 55 |
|  |  | R:5′-TCCTGGTGCAGCTATGTCTG |  |
| 69 | RM321 | F: 5′-CCAACACTGCCACTCTGTTC-3′ | 55 |
|  |  | R:5′-GAGGATGGACACCTTGATCG-3′ |  |
| 70 | RM327 | F: 5′-CTACTCCTCTGTCCCTCCTCTC-3′ | 55 |
|  |  | R:5′-CCAGCTAGACACAATCGAGC-3′ |  |
| 71 | RM331 | F: 5′-GAACCAGAGGACAAAAATGC -3′ | 55 |
|  |  | R:5′-CATCATACATTTGCAGCCAG -3′ |  |
| 72 | RM332 | F: 5′-GCGAAGGCGAAGGTGAAG -3′ | 55 |
|  |  | R:5′-CATGAGTGATCTCACTCACCC-3′ |  |

**Appendix 4: Continued**

| **Serial no.** | **SSR Marker** | **Sequence** | **Tm (°C)** |
| --- | --- | --- | --- |
| 73 | RM333 | F: 5′-GTACGACTACGAGTGTCACCAA-3′ | 55 |
|  |  | R:5′-GTCTTCGCGATCACTCGC-3′ |  |
| 74 | RM339 | F: 5′-GTAATCGATGCTGTGGGAAG-3′ | 55 |
|  |  | R:5′-GAGTCATGTGATAGCCGATATG-3′ |  |
| 75 | RM340 | F: 5′-GGTAAATGGACAATCCTATGGC-3′ | 55 |
|  |  | R:5′-GACAAATATAAGGGCAGTGTGC-3′ |  |
| 76 | RM342 | F: 5′-CCATCCTCCTACTTCAATGAAG-3′ | 55 |
|  |  | R:5′-ACTATGCAGTGGTGTCACCC-3′ |  |
| 77 | RM345 | F: 5′-ATTGGTAGCTCAATGCAAGC-3′ | 55 |
|  |  | R:5′-GTGCAACAACCCCACATG-3′ |  |
| 78 | RM350 | F: 5′- TGATCGTCGCGATTCCCGGC-3′ | 55 |
|  |  | R: 5′- CCCCACCCTGCGCCTCTCCC-3′ |  |
| 79 | RM401 | F: 5′-TGGAACAGATAGGGTGTAAGGG-3′ | 55 |
|  |  | R:5′-CCGTTCACAACACTATACAAGC-3′ |  |
| 80 | RM413 | F: 5′-GGCGATTCTTGGATGAAGAG-3′ | 55 |
|  |  | R: 5′- TCCCCACCAATCTTGTCTTC-3′ |  |
| 81 | RM417 | F: 5′-CGGATCCAAGAAACAGCAG-3′ | 55 |
|  |  | R:5′-TTCGGTATCCTCCACACCTC-3′ |  |
| 82 | RM428 | F: 5′-AACAGATGGCATCGTCTTCC-3′ | 55 |
|  |  | R:5′-CGCTGCATCCACTACTGTTG -3′ |  |
| 83 | RM434 | F: 5′-GCCTCATCCCTCTAACCCTC-3′ | 55 |
|  |  | R:5′-CAAGAAAGATCAGTGCGTGG-3′ |  |
| 84 | RM435 | F: 5′-ATTACGTGCATGTCTGGCTG-3′ | 55 |
|  |  | R:5′-CGTACCTGACCATGCATCTG-3′ |  |
| 85 | RM441 | F: 5′-ACACCAGAGAGAGAGAGAGAGAG -3′ | 55 |
|  |  | R:5′-TCTGCAACGGCTGATAGATG-3′ |  |
| 86 | RM451 | F: 5′-GATCCCCTCCGTCAAACAC -3′ | 55 |
|  |  | R:5′-CCCTTCTCCTTTCCTCAACC -3′ |  |
| 87 | RM455 | F: 5′-AACAACCCACCACCTGTCTC-3′ | 55 |
|  |  | R:5′-AGAAGGAAAAGGGCTCGATC-3′ |  |
| 88 | RM456 | F: 5′-TTGTAGTCCGGGTCGTAACC-3′ | 55 |
|  |  | R:5′-GATAGAATAGGGAGGGGGGG-3′ |  |
| 89 | RM458 | F: 5′-GGTGATCTGCATTGTCAACG-3′ | 55 |
|  |  | R:5′-TGCAATGGATCTAGCGACTG-3′ |  |
| 90 | RM460 | F: 5′-TGATCGACAGCGTTCTTGAC-3′ | 55 |
|  |  | R:5′-GCCTGGCCCACATAATTAAG-3′ |  |

**Appendix 4: Continued**

| **Serial no.** | **SSR Marker** | **Sequence** | **Tm (°C)** |
| --- | --- | --- | --- |
| 91 | RM462 | F: 5′- ACGGCCCATATAAAAGCCTC-3′ | 55 |
|  |  | R: 5′- AAGATGGCGGAGTAGCTCAG-3′ |  |
| 92 | RM465 | F: 5′- GTGCCTCCATCATCATCATC-3′ | 55 |
|  |  | R: 5′- TAGGACAAGCGAAGAAACCG-3′ |  |
| 93 | RM468 | F: 5′-CCCTTCCTTGTTGTGGCTAC-3′ | 55 |
|  |  | TGATTTCTGAGAGCCAACCC-3′ |  |
| 94 | RM469 | F: 5′-AGCTGAACAAGCCCTGAAAG-3′ | 55 |
|  |  | R:5′-GACTTGGGCAGTGTGACATG-3′ |  |
| 95 | RM471 | F: 5′-ACGCACAAGCAGATGATGAG-3′ | 55 |
|  |  | R:5′-GGGAGAAGACGAATGTTTGC-3′ |  |
| 96 | RM480 | F: 5′-GCTCAAGCATTCTGCAGTTG-3′ | 55 |
|  |  | R:5′-GCGCTTCTGCTTATTGGAAG-3′ |  |
| 97 | RM482 | F: 5′- TCTGAAAGCCTGACTCATCG-3′ | 55 |
|  |  | R: 5′- GTCAATTGCAGTGCCCTTTC-3′ |  |
| 98 | RM495 | F: 5′-AATCCAAGGTGCAGAGATGG -3′ | 55 |
|  |  | R:5′-CAACGATGACGAACACAACC -3′ |  |
| 99 | RM498 | F: 5′- AATCTGGGCCTGCTCTTTTC-3′ | 55 |
|  |  | R: 5′- TCCTAGGGTGAAGAAAGGGG-3′ |  |
| 100 | RM500 | F: 5′-GAGCTTGCCAGAGTGGAAAG-3′ | 55 |
|  |  | R:5′-GTTACACCGAGAGCCAGCTC-3′ |  |
| 101 | RM511 | F: 5′- CTTCGATCCGGTGACGAC-3′ | 55 |
|  |  | R: 5′- AACGAAAGCGAAGCTGTCTC-3′ |  |
| 102 | RM512 | F: 5′- CTGCCTTTCTTACCCCCTTC-3′ | 55 |
|  |  | R: 5′- AACCCCTCGCTGGATTCTAG-3′ |  |
| 103 | RM515 | F: 5′-TAGGACGACCAAAGGGTGAG-3′ | 55 |
|  |  | R:5′-TGGCCTGCTCTCTCTCTCTC-3′ |  |
| 104 | RM529 | F: 5′-CCCTCCCTTCTGTAAGCTCC-3′ | 55 |
|  |  | R:5′-GAAGAACAATGGGGTTCTGG-3′ |  |
| 105 | RM543 | F: 5′- CTGCTGCAGACTCTACTGCG-3′ | 55 |
|  |  | R: 5′- AAATATTACCCATCCCCCCC-3′ |  |
| 106 | RM546 | F: 5′- CATGGCCTTGTGTATGCATC-3′ | 55 |
|  |  | R: 5′- ATGCAGAGGATTGGCTTGAG-3′ |  |
| 107 | RM553 | F: 5′-AACTCCACATGATTCCACCC-3′ | 55 |
|  |  | R:5′-GAGAAGGTGGTTGCAGAAGC-3′ |  |
| 108 | RM555 | F: 5′-TTGGATCAGCCAAAGGAGAC-3′ | 55 |
|  |  | R:5′-CAGCATTGTGGCATGGATAC-3′ |  |

**Appendix 4: Continued**

| **Serial No.** | **SSR Marker** | **Sequence** | **Tm (°C)** |
| --- | --- | --- | --- |
| 109 | RM557 | F: 5′-GTGGCGAGATCTATGTGGTG-3′ | 55 |
|  |  | R:5′-GCTTTGTGTGTGTGTGTGTG-3′ |  |
| 110 | RM563 | F: 5′-CGACCCTAGGGTTTCTCC-3′ | 55 |
|  |  | R:5′-CTCGACGTCGTGGAAAGC-3′ |  |
| 111 | RM564 | F: 5′-CATGGCCTTGTGTATGCATC-3′ | 55 |
|  |  | R:5′-ATGCAGAGGATTGGCTTGAG-3′ |  |
| 112 | RM570 | F: 5′-GTTCTTCAACTCCCAGTGCG-3′ | 55 |
|  |  | R:5′-TGACGATGTGGAAGAGCAAG-3′ |  |
| 113 | RM585 | F: 5′-CAGTCTTGCTCCGTTTGTTG-3′ | 55 |
|  |  | R:5′-CTGTGACTGACTTGGTCATAGG-3′ |  |
| 114 | RM588 | F: 5′-GTTGCTCTGCCTCACTCTTG-3′ | 55 |
|  |  | R:5′-AACGAGCCAACGAAGCAG-3′ |  |
| 115 | RM1106 | F: 5′-CGGAAAGTGAATCGGAGAAC-3′ | 55 |
|  |  | R:5′-GCACCACGCTAAGCTAAACC-3′ |  |
| 116 | RM1109 | F: 5′-TCAAAATCACGTGTATGTAAGC-3′ | 55 |
|  |  | R:5′-TTTACAAAGGACAGAGGGC-3′ |  |
| 117 | RM1237 | F: 5′-CTCCGCGAGCTTTAGAAGAG-3′ | 55 |
|  |  | R:5′-CACATACTCTGGCTCTCCCG-3′ |  |
| 118 | RM1359 | F: 5′- AACGAATTCTATTTTGCGTC-3′ | 55 |
|  |  | R: 5′- TTCTTCTCATTTCAATTCGC-3′ |  |
| 119 | RM1364 | F: 5′-AAGAAATTCAAAACACATGA-3′ | 55 |
|  |  | R:5′-AAAACATCTACTTTGATCCA-3′ |  |
| 120 | RM2655 | F: 5′-AAATTGAATTGAGCATATTA-3′ | 55 |
|  |  | R:5′-ATCAAAAGTATTTTGTGTTG-3′ |  |
| 121 | RM3123 | F: 5′-ATTTCCCACACATCTCGCTG-3′ | 50 |
|  |  | R:5′-GTGTCGCCGGTCAAGAAC-3′ |  |
| 122 | RM3131 | F: 5′-CTCTGCACCCTGTTCACATG-3′ | 55 |
|  |  | R:5′-CCCAATGGAATATCAGGTGG-3′ |  |
| 123 | RM3134 | F: 5′-GCAGGCACAAAAGCAAAGAG-3′ | 50 |
|  |  | R:5′-AGGTGAAGGTGCATTGTGTG-3′ |  |
| 124 | RM3187 | F: 5′-TCCCCACATCGTGTCGTC-3′ | 50 |
|  |  | R:5′-TTTTTCCCCTTCTACCCTCG-3′ |  |
| 125 | RM3353 | F: 5′-AATGGTCGCCTCTCTCTCTG-3′ | 50 |
|  |  | R:5′-GCTGGCATTGACCGTGTC-3′ |  |
| 126 | RM3355 | F: 5′-CATATGCAATTGATGTTTCG-3′ | 55 |
|  |  | R:5′-TTAATTCCTTGGTCTCAAATG-3′ |  |
| 127 | RM3726 | F: 5′-CACACACATCGCTCGGTC-3′ | 50 |
|  |  | R:5′-GATGTGGAGGTCGATGGC-3′ |  |

**Appendix 4: Continued**

| **Serial No.** | **SSR Marker** | **Sequence** | **Tm (°C)** |
| --- | --- | --- | --- |
| 128 | RM3766 | F: 5′-TTATAGAGCCAACACAACGG-3′ | 55 |
|  |  | R:5′-ATCGATCTCTCTCCTGGAAA-3′ |  |
| 129 | RM3872 | F: 5′-GGAAGAAAGGATCTATATCA-3′ | 55 |
|  |  | R:5′-TACGATTTGTTTAAGTTCAA-3′ |  |
| 130 | RM5340 | F: 5′-TGGGCCCTAAGTCATATTGC-3′ | 50 |
|  |  | R:5′-ACCCAACGAGATGTACCTCG-3′ |  |
| 131 | RM5341 | F: 5′-TGCATTTTCCATACAATACG-3′ | 55 |
|  |  | R:5′-ATTTGATACATGGACGATGC-3′ |  |
| 132 | RM5,531 | F: 5′-TTTGTGTTGGTAAGTTGCTTC-3′ | 55 |
|  |  | R:5′-TTAAGGAGAGTGTTTTCTTTTCTC-3′ |  |
| 133 | RM5639 | F: 5′-GGAAGAACAGAGTTGCTCGG-3′ | 55 |
|  |  | R:5′-GTGCCATTTATTTCCGTCCC-3′ |  |
| 134 | RM5,742 | F: 5′-GGCGAGCGATCCTCAAAC-3′ | 55 |
|  |  | R:5′-GTTTACTCACGCTCTGCCAG-3′ |  |
| 135 | RM6250 | F: 5′-AACCTACGTTACCCTGCACG-3′ | 50 |
|  |  | R:5′-GGCTCATGAGTTTCAGAGGC-3′ |  |
| 136 | RM6293 | F: 5′-GGCTCGATCGATTGGATTC-3′ | 55 |
|  |  | R: 5′- TCACTAAAACGCGTTACGGG-3′ |  |
| 137 | RM7018 | F: 5′-CATCGTTGACCGCTGCTC-3′ | 50 |
|  |  | R:5′-AATAAACAGCACGTGCTCCC-3′ |  |
| 138 | RM7376 | F: 5′-TCACCGTCACCTCTTAAGTC-3′ | 50 |
|  |  | R:5′-GGTGGTTGTGTTCTGTTTGG-3′ |  |
| 139 | RM8225 | F: 5′-ATGCGTGTTCAGAAATTAGG -3′ | 55 |
|  |  | R:5′-TTGTTGTATACCTCATCGACAG-3′ |  |
| 140 | RM10000 | F: 5′-AATCCAGATGTGCGCCAACC-3′ | 55 |
|  |  | R:5′-GAGTGAAGCCAGCTCCTCAACC-3′ |  |
| 141 | RM10001 | F: 5′-CAATCACCCTCACCCTCTTATATGC-3′ | 55 |
|  |  | R:5′-CCGCTGTGAACAACAATCATGC-3′ |  |
| 142 | RM10017 | F: 5′-GATTCCTCATTCCATGTCTTGC-3′ | 55 |
|  |  | R:5′-AATTGTATGCCTGCATCTGTCC-3′ |  |
| 143 | RM10018 | F: 5′-ACTAGTACACCTCAACTTCACTCC-3′ | 55 |
|  |  | R:5′-CCTTTAGTTTGCTTGTGACC-3′ |  |
| 144 | RM10022 | F: 5′-CCTCCATAGAGTAAGGTTTGCATGG-3′ | 55 |
|  |  | R:5′-CCTCCTCCTCTGTCTTTCTCTGC-3′ |  |
| 145 | RM10026 | F: 5′-AATCGATCCAAACAAGGCAAGC-3′ | 55 |
|  |  | R:5′-TCACTAATAACCTGACGACCTCTGC-3′ |  |
| 146 | RM16655 | F: 5′-CCTTGGAAGCTGGAACTTCACC-3′ | 55 |
|  |  | R:5′-GGCTCTTAGGTTAGATCCCACACG-3′ |  |
| 147 | RM23835 | F: 5′-TTCCGCTGTTTCTCTTCTTGTGC-3′ | 55 |
|  |  | R:5′-CTGGTTCTGCTGGTTCTGTAGTTGG-3′ |  |
